# Evaluation of DeepSeek with a RAG-based knowledge base in preoperative assessment for arthroplasty to improve the decision-making accuracy of junior physicians

**DOI:** 10.1186/s42836-026-00437-z

**Published:** 2026-10-13

**Authors:** Haibo Wu, Mingzhuang Hou, Gongkai Chen, Zhenlin Wen, Changao Yang, Songlin Li, Xiangfei Meng, Peilai Liu, Qunshan Lu

**Affiliations:** 1 Department of Orthopedics, Qilu Hospital of Shandong University, Jinan, 250012 China; 2 National Supercomputer Center in Tianjin, Tianjin, 300457 China

**Keywords:** Large language models, DeepSeek, Preoperative assessment, Knowledge base

## Abstract

**Background:**

Patients undergoing elective joint replacement are mostly elderly individuals with multiple chronic diseases. These patients have a higher risk, so preoperative assessment for joint replacement is vital for surgical safety but remains challenging for junior physicians. This study aimed to evaluate the decision-making performance of different versions of DeepSeek (Chat and R1) and their knowledge base (KB)-enhanced schemes in perioperative risk assessment and their impact on junior physicians’ decision-making quality.

**Methods:**

A total of 292 anonymized medical records from joint replacement patients were retrospectively analyzed. A professional knowledge base was constructed using 21 perioperative management guidelines and retrieval-augmented generation (RAG) technology. The performance of four models (DeepSeek-Chat, DeepSeek-Chat + KB, DeepSeek-R1, and DeepSeek-R1 + KB) was compared in ASA scoring, RCRI scoring, surgical tolerance judgment, and risk prediction. These indicators are related to specific decisions in joint replacement surgery, including preoperative optimization before elective TKA/THA, mitigation of infection risk, planning of VTE prophylaxis, and indication for postponing elective arthroplasty, etc. Senior physicians evaluated model performance using a Likert scale. A 5 × 5 balanced Latin square crossover design was used to assess the impact of model assistance on junior physicians’ clinical decision-making.

**Results:**

No significant difference was found in ASA scoring accuracy (*p* > 0.05). For RCRI scoring and risk stratification, DeepSeek-R1 models (79.1%–79.7%) outperformed Chat models (56.5%–66.0%, *p* < 0.05). In surgical tolerance assessment, DeepSeek-R1 + KB showed relatively better performance, with significantly better predictive precision (*p* = 0.0284) and a lower missed diagnosis rate (*p* = 0.005) compared to the Chat models. The R1 series also showed higher predictive efficiency (87.2%–86.4% vs. 63.6%–61.8%, *p* < 0.05). The R1 models showed better performance than Chat models in medical comprehension, retrieval accuracy, and reasoning logic. After adjusting for physician variability and patient case mix, the DeepSeek-R1 + KB showed a statistically significant improvement over independent decision-making (OR = 1.58, *p* = 0.004), as well as the DeepSeek-R1 and Chat series.

**Conclusion:**

The DeepSeek-R1 model, with chain-of-thought reasoning, shows a marginal advantage over the Chat version in preoperative assessment for joint replacement. As a cost-effective clinical auxiliary tool, its integration with a professional knowledge base scheme can optimize the decision-making quality of junior physicians, particularly in terms of providing helpful and comprehensive decision support and providing novel insights for individualized assessment in the context of precision medicine.

**Supplementary Information:**

The online version contains supplementary material available at https://doi.org/10.1186/s42836-026-00437-z.

## Introduction

Joint replacement surgery is the gold standard for the treatment of end-stage joint diseases and has become a key measure to improve patients’ quality of life [1–3]. However, with the intensification of population aging, the cohort of patients undergoing joint replacement is increasingly trending toward advanced age. These patients are often accompanied by multiple comorbidities, such as cardiovascular diseases, diabetes mellitus, and obesity, making preoperative assessment particularly complex. Accurate preoperative assessment is crucial for evaluating surgical tolerance, predicting operative risks, and optimizing perioperative management [4].

The difficulty of preoperative assessment lies in the need to comprehensively integrate multi-dimensional patient information, including medical history, physical examination findings, laboratory test results, and imaging data [5]. Such information is characterized by substantial inter-individual variability and complex correlations, requiring physicians to identify key elements for evidence-based decision-making. For instance, factors such as patient age, body weight, cardiovascular status, and prior surgical history can all influence surgical risks. Nevertheless, the heterogeneity of clinical presentations across different patients poses a challenge to the accuracy of assessment outcomes. Traditional preoperative assessment methods rely heavily on physicians’ clinical experience. While senior practitioners can deliver relatively accurate evaluations, this approach remains prone to subjectivity and limitations for junior physicians. Particularly when dealing with elderly patients with multiple comorbidities, junior physicians may encounter the dilemma of information overload, struggling to synthesize all relevant factors effectively.

With the rapid advancement of artificial intelligence (AI) technology, the application potential of large language models (LLMs) in the medical field has gradually emerged [6–8], especially demonstrating immense prospects in preoperative assessment [9]. LLMs are capable of processing and analyzing massive volumes of medical data, with the potential to generate personalized assessment recommendations based on comprehensive data synthesis. More importantly, LLMs can assist physicians in simplifying complex medical terminology during patient consultations, providing plain-language explanations that enable patients to gain a clearer understanding of their health conditions [10]. Through real-time feedback and intelligent recommendations, LLMs are expected to provide decision support for physicians, facilitating more precise and comprehensive preoperative assessments. Particularly in the management of elderly patients with multiple comorbidities, LLMs have the potential to break through the limitations of traditional assessment methods that are highly dependent on physician experience, thus improving the efficiency and accuracy of evaluations. Therefore, exploring the application of LLMs in preoperative assessment not only contributes to enhancing the quality of medical services but also offers novel ideas and tools for future medical decision-making.

As AI technology advances at a rapid pace, various LLMs have demonstrated tremendous application potential in the medical domain. Among these models, DeepSeek not only maintains a benchmark-level performance comparable to top models, but also significantly optimizes the computing efficiency and deployment costs [11]. However, general-purpose LLMs may still suffer from limitations such as generative hallucinations or delayed updates of professional guideline knowledge when dealing with highly specialized clinical scenarios. Therefore, the integration of domain-specific professional knowledge bases is recognized as an effective approach to enhance the decision-making accuracy of models [12]. Currently, systematic evaluations are still lacking regarding the performance differences among various DeepSeek model architectures in processing complex clinical logic, as well as the actual impact of knowledge base-enhanced strategies on clinical decision-making quality.

The purpose of this study was to evaluate the application efficacy of different versions of DeepSeek and their knowledge base integration strategies in perioperative preoperative assessment for joint replacement surgery. It aimed to clarify the accuracy of these models in judging surgical tolerance and predicting perioperative risks, while simultaneously exploring their auxiliary value in supporting the clinical decision-making of junior physicians.

## Method

### Study subjects

**Fig. 1:**
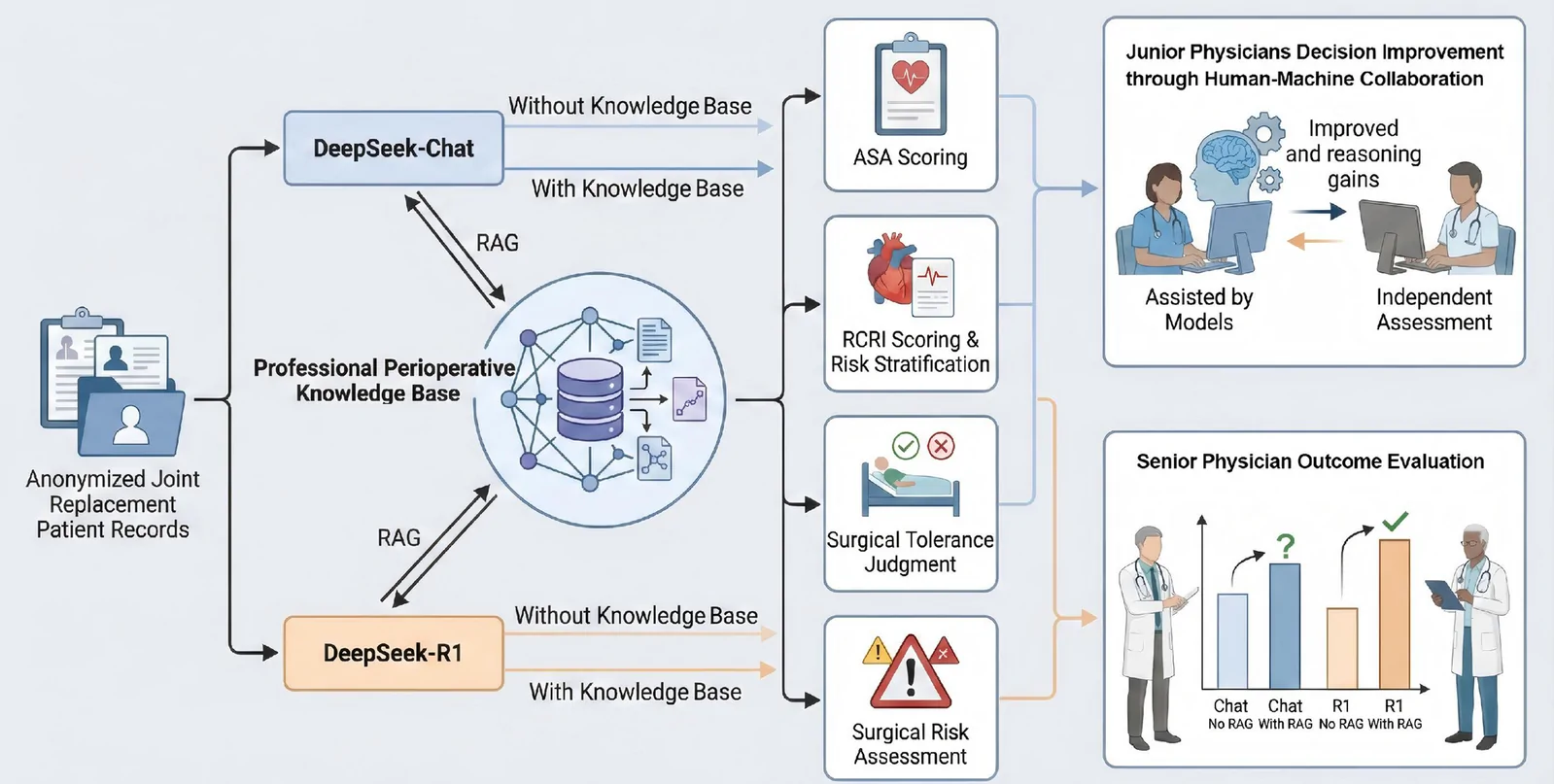
Schematic illustration of the study workflow. The study utilized anonymized medical records of patients undergoing joint replacement as the dataset. Two core large language models, DeepSeek-Chat and DeepSeek-R1, were evaluated under two conditions: without a knowledge base and with a Retrieval-Augmented Generation-enhanced professional knowledge base comprising 21 authoritative perioperative guidelines. This created four experimental groups to perform preoperative tasks, including ASA scoring, RCRI scoring & risk stratification, surgical tolerance judgment, and surgical risk assessment. The evaluation framework consisted of two parts: (1) Model performance evaluation, where outputs were validated by senior physicians (bottom right); and (2) Human–machine collaboration assessment, employing a crossover design to quantify the improvement in decision-making accuracy of junior physicians when assisted by the models versus independent assessment (top right)

This study was approved by the Ethics Committee of Qilu Hospital of Shandong University (Approval No. [KYLL-2025(ZM)-224]). This study adhered to the requirements of the DECIDE-AI guidelines and provided a complete DECIDE-AI checklist, which can be found in Supplementary Material 4. A retrospective analysis was conducted on the medical records of 323 hospitalized patients scheduled for joint replacement surgery between January and August 2025. All patients met the surgical indications for total hip or total knee arthroplasty. After excluding 31 cases with incomplete records, anonymized data from 292 patients were included. Details are provided in Fig. 1, with demographic characteristics summarized in Table 1. All records underwent strict de-identification by researchers. Extracted clinical data included gender, age, BMI, chief complaints, medical history, physical examination findings, and auxiliary test results, providing multi-dimensional clinical context for the models.

**Table 1:** Demographic characteristics statistics

Characteristics	N = 292
Age (years)	59.0 ± 16.0
BMI (Kg/m^2^)	26.7 ± 3.4
Gender (%)	
Female	206 (70.5)
Male	86 (29.5)
Surgical Status (%)	
Underwent Surgery	257 (88.0)
Did Not Undergo Surgery	35 (12.0)
Surgical Procedure (%)	
TKA	137 (53.3)
THA	120 (46.7)

Percentages for TKA and THA are based on the 257 patients who actually underwent surgery, not the full cohort of 292 patients

BMI: Body Mass Index; TKA: Total Knee Arthroplasty; THA: Total Hip Arthroplasty

### Model configuration and knowledge base construction

The DeepSeek-Chat and DeepSeek-R1 online models (V3.2-Exp, 2025-09-29, DeepSeek AI Co., Ltd., Hangzhou, China) served as the core engines. A custom workflow was developed using the open-source LLM platform Dify (Version 1.8.1, 2025-09-03, Yuling AI Tech Co., Ltd., Suzhou, China) [13]. All models were invoked through DeepSeek’s API with the web search function disabled throughout inference, while the Dify platform and its knowledge base were deployed locally, and all inference parameters remained fixed to ensure experimental reproducibility. API-based models are subject to potential version updates, and we recommend that future studies consider local deployment or pinned API versions for enhanced reproducibility. 21 authoritative perioperative management guidelines were retrieved from PubMed and UpToDate, covering key areas such as cardiopulmonary assessment, comorbidity management, thromboprophylaxis, and infection control. A domain-specific knowledge base was constructed using 21 perioperative management guidelines. Supplementary Material 1 provides the full list of guidelines, including titles, publication years, issuing organizations, and specification of whether each guideline is arthroplasty-specific (*n* = 6) or general perioperative (*n* = 15). Guidelines were selected if they were: (a) published within the last 10 years, (b) issued by a nationally or internationally recognized professional society, or published on UpToDate websites, and (c) directly relevant to perioperative care of arthroplasty patients (including general guidelines applicable to this population). All guidelines were converted into plain text, split into semantic chunks (chunk size: 500 characters with 50-character overlap), and embedded using the default embedding model (Qwen3-Embedding-8B) provided by Dify (version 1.8.1). The resulting vectors were stored in a local vector database. During retrieval, the top-3 most semantically similar chunks were retrieved for each user query and appended to the LLM prompt. No manual relevance filtering was performed, as the retrieval quality was inspected on a pilot set of 20 queries and deemed acceptable (> 90% relevant).

### Comparison between different LLMs

This study tested four DeepSeek models: DeepSeek-Chat, DeepSeek-Chat + KB, DeepSeek-R1, and DeepSeek-R1 + KB. All models ran offline, with workflows developed on the Dify platform. [14, 15]. The basic models without knowledge base integration followed a simple workflow: Input—LLM—Output. The enhanced models with knowledge base integration followed: Input—Knowledge Base Retrieval—LLM—Output. The Instruction-Context-Input–Output (ICIO) prompt framework was used for interactions. [16]. The model acted as the “Attending Orthopedic Surgeon” to complete the American Society of Anesthesiologists (ASA) and revised cardiac risk index (RCRI) scoring, RCRI risk stratification, provide rationale explanations, assess surgical tolerance, decide on surgery postponement, and suggest management targets (e.g., infection control, blood pressure and glycemic regulation). The complete prompt template can be found in the new Supplementary Material 3.

### Evaluation metrics

#### ASA score, RCRI score, and RCRI risk stratification

The model’s output results were compared with the ASA score, RCRI score, and RCRI risk stratification documented in the medical records [17]. These scores were obtained from the preoperative assessment sheet, completed by anesthesiologists.

The ASA score evaluates preoperative health and surgical risk [18], classifying patients into six grades. Grade I denotes healthy individuals, Grade V represents patients with extremely high anesthetic risk, and Grade VI refers to brain-dead patients for organ donation. Because ASA classification is known to be subjective, we evaluated the interobserver reliability of the reference standard. Two independent senior anesthesiologists (each with > 10 years of experience, blinded to the original scores and to each other) re-assigned ASA scores for a random subset of 50 patients using the same clinical data. Fleiss’ kappa was calculated to quantify agreement among the original anesthesiologist and the two independent reviewers.

The RCRI score assesses the risk of major cardiovascular complications during non-cardiac surgery [19], based on six risk factors: high-risk surgery, ischemic heart disease, chronic heart failure, cerebrovascular disease, insulin-dependent diabetes, and preoperative serum creatinine > 2.0 mg/dL. Each factor scores 1 point, with a total score ranging from 0 to 6. Scores of 0–1 indicate low risk, 2 points moderate risk, and ≥ 3 points high risk.

#### The judgment of surgical tolerance

To assess the models’ decision-making in surgical tolerance, the actual outcome (whether the patient underwent surgery) was used as the gold standard. The surgical tolerance decision was made by a senior orthopedic surgeon together with an anesthesiologist, based on routine clinical practice. The model’s judgment was compared with this outcome, and a confusion matrix was applied for classification evaluation [20]. “Tolerable for surgery” was considered positive, while “Intolerable for surgery or surgery needs to be postponed” was negative. The diagnostic performance of the models in determining surgical tolerance was quantified using four metrics: True Positive Rate (TPR) = True Positive (TP)/[TP + False Negative (FN)], indicating sensitivity for identifying surgical candidates; True Negative Rate (TNR) = True Negative (TN)/[TN + False Positive (FP)], representing specificity in excluding high-risk cases; False Positive Rate (FPR) = FP/(TN + FP), measuring the risk of misdiagnosing high-risk patients as eligible; and False Negative Rate (FNR) = FN/(TP + FN), quantifying the risk of missing surgical opportunities.

#### The assessment of surgical risks

Surgical risk assessment followed a standardized process. Three orthopedic professors defined 10 risk categories based on perioperative management guidelines. The experts predicted risks using patient data, established gold standard annotations [21], and counted positive cases for each risk category. Among them, three senior orthopedic professors independently reviewed all 292 anonymized cases. They were blinded to model outputs and to actual postoperative outcomes. For each case and each risk category, experts judged whether the patient was at “high risk” (positive) or “low risk” (negative). Cases with disagreement among the three experts were resolved by majority vote (≥ 2/3). The final gold standard annotation consisted of the majority consensus. Clinical data were then input into the four groups of LLMs, and each model’s prediction accuracy was evaluated by comparing the number of positive cases identified by the model to the total number of expert-annotated positive cases. For each risk category, the model’s prediction accuracy rate was calculated as: Prediction accuracy rate = (Number of positive cases predicted by the model)/(Number of positive cases annotated by the expert panel) × 100%. This metric reflects the model’s ability to identify the overall volume of high-risk patients at the group level, which is informative for preoperative risk stratification and resource planning.

#### Model performance evaluation

An evaluation team of five senior orthopedic surgeons (with over 3 years of experience) was formed. The evaluation followed a blinded process: five evaluators received responses from the four models for the same clinical case without knowing which model produced each response. For each medical record generated by each model, five senior doctors conducted a professional discussion and then provided a common result. The panel assessed the models’ technical performance based on correct comprehension, retrieval, and reasoning in medical text processing [22]. Clinical utility was evaluated by senior surgeons using a 5-point Likert scale across four dimensions: correctness, helpfulness, completeness, and adaptability [23–25]. These grading criteria were jointly formulated and agreed upon by all the review team members before the assessment, aiming to achieve standardized evaluations among the assessors. Detailed grading criteria are provided in Supplementary Material 2.

#### Evaluation of the gain in human–machine collaborative decision-making

To quantify the decision-making improvement from DeepSeek models, a 5 × 5 balanced Latin square crossover design was used. The 292 anonymized medical records were randomly divided into 5 case sets (Set A–E, 58–59 cases each) [26]. Each set was evaluated by five junior orthopedic physicians (with less than 3 years of experience) using two approaches: without LLM assistance and with one of the four DeepSeek models. Evaluation indicators included ASA score, RCRI score, RCRI risk stratification, and surgical tolerance. To ensure reliability, each physician experienced both modes (independent and model-assisted), and assessment methods were evenly distributed across case sets to minimize the impact of clinical ability differences and case variability. The auxiliary value of the models was assessed by comparing the accuracy of junior physicians between the two approaches.

### Data analysis

All data analyses were performed using IBM SPSS 27.0.1 (SPSS, Chicago, IL, USA) and GraphPad Prism 10.4.0 (GraphPad Software, San Diego, California, USA). Quantitative data were expressed as mean ± standard deviation, and the Shapiro‒Wilk test assessed normality. For normally distributed data, one-way analysis of variance (ANOVA) was used, with post hoc comparisons if significant. For non-normally distributed data, the Kruskal‒Wallis test was applied. Categorical data were presented as percentages and frequencies, with the chi-square test or Fisher’s exact test for group comparisons. The effect of LLM assistance on junior physicians’ accuracy was analyzed using a generalized linear mixed model (GLMM) with a binary distribution and logit link function. A significance level of α = 0.05 was set, and *p* < 0.05 was considered statistically significant.

## Results

### The accuracy rates of ASA score, RCRI score, and RCRI risk stratification

**Fig. 2:**
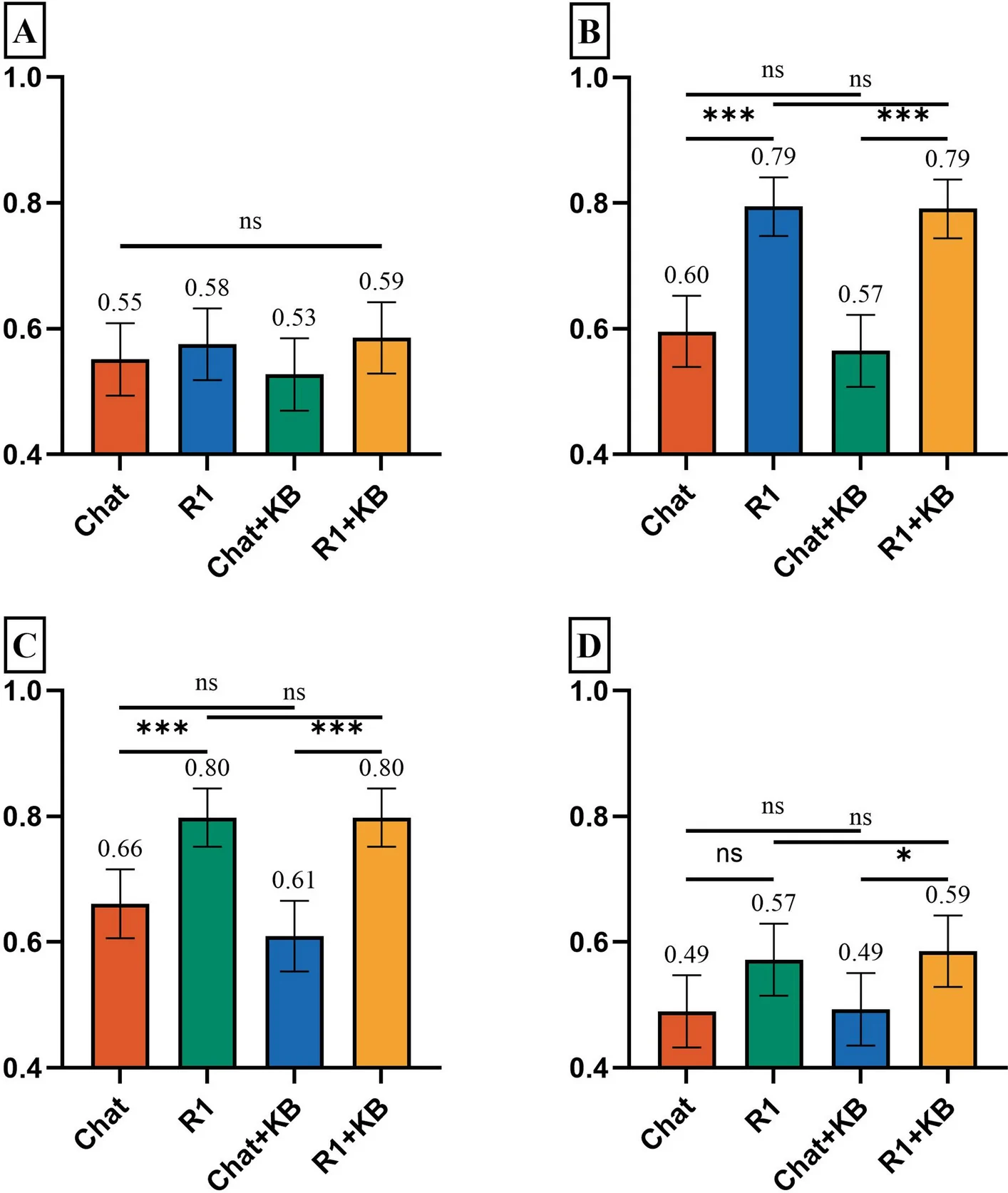
Objective evaluation results of different versions of the DeepSeek model (*p < 0.05; **p < 0.01; ***p < 0.001; Error bars indicate 95% confidence intervals). **A** ASA score accuracy rate. **B** RCRI score accuracy rate. **C** RCRI risk stratification accuracy rate. **D** Surgical judgment accuracy rate

Among the 50 cases randomly selected for interobserver analysis, Fleiss’ kappa for ASA scores among the original anesthesiologist and two independent reviewers was 0.473 (95% CI 0.243–0.620), indicating moderate agreement. This confirms substantial subjectivity in the reference standard. There was no statistically significant difference in the accuracy of ASA scoring among the four models (*p* > 0.05), with DeepSeek-R1 + KB achieving the optimal performance (58.6%), as detailed in Fig. 2A. Overall, the R1 series outperformed the Chat series in accuracy; the integration of the knowledge base exerted significantly different impacts on models with different architectures: the performance of DeepSeek-R1 showed a slight improvement after integrating the knowledge base, whereas the accuracy of Chat declined instead (55.1% vs. 52.7%). In terms of RCRI scoring and risk stratification, the R1 series demonstrated remarkable performance advantages, with its scoring accuracy (approximately 79.4%) and stratification accuracy (79.7%) both significantly surpassing those of the Chat series (56.5%–66.0%, *p* < 0.05). Pairwise statistical comparisons indicated a significant difference between the R1 and Chat series (*p* < 0.05). In contrast, the integration of the knowledge base within the same architecture did not yield a significant performance improvement, and the accuracy of the Chat series even exhibited a slight downward trend after incorporating the knowledge base (see Figs. 2B/C).

### The accuracy rate of surgical tolerance judgment

In terms of surgical tolerance judgment (Fig. 2D, Table 2), there were statistically significant differences among the four models in both accuracy (*p* = 0.0284) and FNR (*p* = 0.005). Among them, DeepSeek-R1 + KB exhibited the optimal performance (accuracy: 58.6%, TPR: 59.1%), and its ability to seize surgical opportunities and reduce missed diagnosis risks (FNR: 40.9%) was significantly superior to that of the Chat series (*p* < 0.05). Although the integration of the knowledge base brought a slight performance improvement (0.3%–1.4%), the model architecture (R1 vs. Chat) was the key factor leading to the generational gap in diagnostic efficacy. However, there were no statistically significant differences in specificity and FPR among the four models (*p* = 0.736), indicating that all models currently remain at the same level in the safety evaluation of excluding high-risk surgical cases.

**Table 2:** Confusion matrix and classification accuracy for surgical tolerance assessment

Model	TPR (%)	TNR (%)	FPR (%)	FNR (%)
DeepSeek-Chat	47.1	62.9	37.1	52.9
DeepSeek-R1	58.4	48.6	51.4	41.6
DeepSeek-Chat + KB	47.5	62.9	37.1	52.5
DeepSeek-R1 + KB	59.1	54.3	45.7	40.9

TPR: True Positive Rate; TNR: True Negative Rate; FPR: False Positive Rate; FNR: False Negative Rate

### Surgical risk prediction

**Fig. 3:**
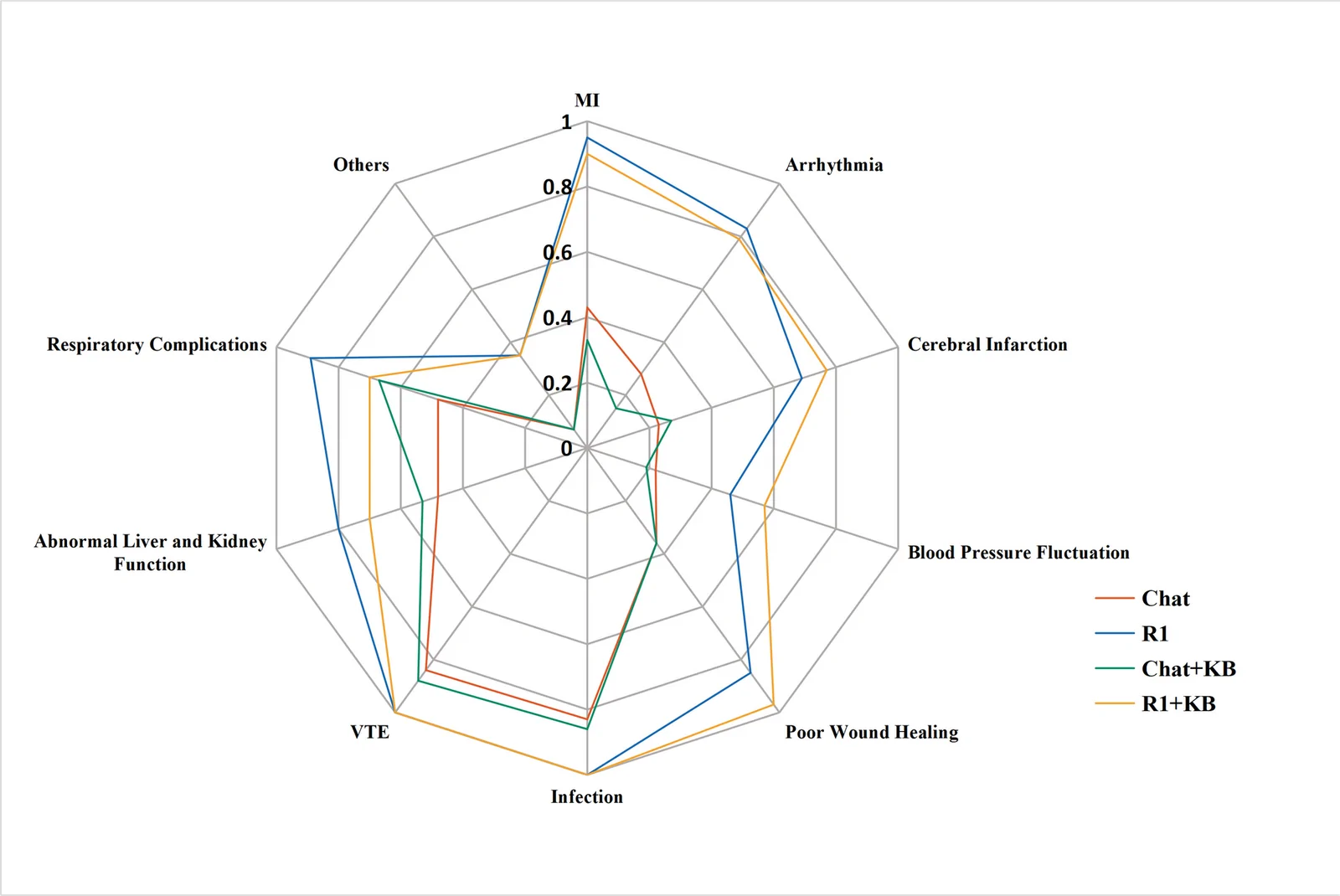
Risk prediction radar charts of different versions of the DeepSeek model. The radial distance in the chart represents the model’s assessment accuracy for the corresponding complication, scaled from 0 to 1, with 1 indicating the best performance. MI: Myocardial Infarction; VTE: Venous Thromboembolism; Others: Other Risks

The R1 series models were significantly superior to the Chat series models in risk prediction efficacy: the average prediction accuracy rates of the DeepSeek-R1 + KB group and the DeepSeek-R1 basic group reached 87.2% and 86.4%, respectively, while those of the DeepSeek-Chat + KB group and the DeepSeek-Chat basic group were only 63.6% and 61.8%. Among specific risk types, both the DeepSeek-R1 and DeepSeek-R1 + KB models achieved 100% prediction accuracy for “Infection” and “VTE”, the DeepSeek-R1 + KB group achieved an accuracy rate exceeding 90% in predicting the risks of “MI” and “Poor Wound Healing”; the DeepSeek-R1 basic group had an accuracy rate over 85% in predicting the risks of “MI” and “Respiratory Complications”. In contrast, the Chat series models only achieved an accuracy rate > 80% in predicting the risks of “Infection” and “VTE”. The four groups of models showed the highest consistency in predicting “Infection” and “VTE”, while their ability to predict “Other risks” was generally weak (with the prediction accuracy rate not exceeding 35% in all groups). The specific ratios are shown in Fig. 3.

### Model performance evaluation

The R1 series demonstrated significantly higher accuracy than the Chat series in three dimensions: comprehension, retrieval, and reasoning (*p* < 0.05). Among all groups, the DeepSeek-R1 + KB group exhibited the optimal comprehensive performance, with its three indicators (69.4%, 82.3%, and 66.3%) showing significant improvements compared with its base model (*p* < 0.05). In contrast, the integration of the knowledge base did not yield statistically significant enhancements for the Chat series (*p* > 0.05).

**Fig. 4:**
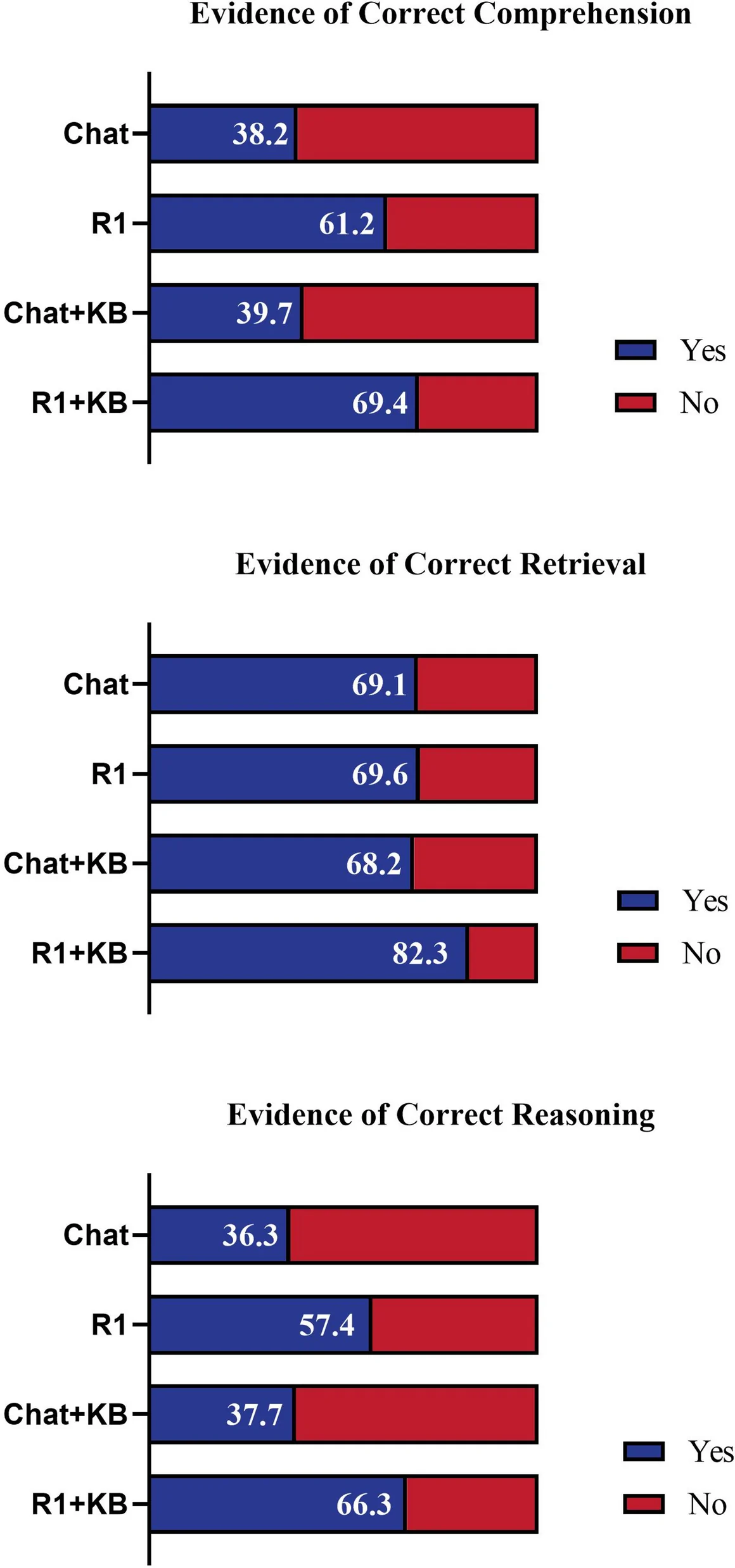
Performance evaluation of different versions of the DeepSeek model. The blue segment (Yes) represents the proportion of correct outcomes for the model in the corresponding dimension, while the red segment (No) represents the proportion of incorrect outcomes; the numerical values indicate specific percentages

Overall, all models showed robust performance in the retrieval dimension (with accuracy rates all > 68%). Nevertheless, reasoning capability remains a universal constraint for models across all current architectures; even the top-performing DeepSeek-R1 + KB group only achieved a reasoning accuracy of 66.3% (see Fig. 4).

### Clinical reference value evaluation

As shown in Table 3, significant differences among the four model groups were observed in the Helpfulness (*p* < 0.05) and Completeness (*p* < 0.05) dimensions. In both dimensions, the DeepSeek-R1 and DeepSeek-R1 + KB models achieved higher upper-quartile scores (up to 5.0) compared with the DeepSeek-Chat and DeepSeek-Chat + KB groups, suggesting that the reasoning-enhanced models were perceived as more helpful and more comprehensive by senior surgeons. No significant differences were found in Correctness or Adaptiveness, nor in the overall total score (all *p* > 0.05). Therefore, any interpretation of superior clinical reference value for the R1 series should be strictly restricted to the Helpfulness and Completeness dimensions, as these were the only metrics showing statistically significant improvements.

**Table 3:** Evaluation by senior orthopedic doctors of the clinical reference value of the response results of different versions of the DeepSeek model

Model	Correctness	Helpful	Completeness	Adaptiveness	Total
Chat	3.00 (2.00, 4.75)	2.00 (2.00, 4.75)	3.00 (3.00, 4.00)	3.00 (2.00, 4.75)	3.00 (2.00, 4.00)
R1	3.00 (2.00, 5.00)	3.00 (2.00, 5.00)	3.00 (3.00, 5.00)	3.00 (2.00, 5.00)	3.00 (2.00, 5.00)
Chat + KB	3.00 (2.00, 4.00)	2.00 (2.00, 4.00)	3.00 (3.00, 4.00)	3.00 (2.00, 4.00)	3.00 (2.00, 4.00)
R1 + KB	3.00 (2.00, 5.00)	3.00 (2.00, 5.00)	3.00 (3.00, 5.00)	3.00 (2.00, 5.00)	3.00 (3.00, 5.00)
	*p* > 0.05	*p* < 0.05	*p* < 0.05	*p* > 0.05	*p* < 0.05

Data are presented as median (interquartile range, IQR). Scores were based on a 5-point Likert scale. P values were calculated using the Kruskal–Wallis test for overall group comparison. IQR = P25–P75

### Evaluation of the gain in human–machine collaborative decision-making

The GLMM revealed a significant main effect of assistance condition on junior physicians’ overall assessment accuracy (F(4, 5828) = 9.802, *p* < 0.001). Pairwise comparisons from the fixed coefficients confirmed that DeepSeek-R1 + KB significantly outperformed the LLMs-free (OR = 1.58, 95% CI 1.16–2.15, *p* = 0.004), DeepSeek-Chat (OR = 2.51, 95% CI 1.86–3.39, *p* < 0.001), DeepSeek-Chat + KB (OR = 1.40, 95% CI 1.02–1.90, *p* = 0.035) and DeepSeek-R1 (OR = 1.82, 95% CI 1.34–2.47, *p* < 0.001). No significant period effect was detected (F (4, 5828) = 0.157, *p* = 0.960), indicating the absence of learning or order effects across the five assessment rounds. A significant task effect was observed (F(3, 5828) = 43.089, *p* < 0.001), reflecting the inherent difficulty differences among the four clinical tasks, with ASA scoring being the most challenging (coefficient = 0.719, *p* < 0.001). Details are shown in Tables 4 and 5.

**Table 4:** Fixed effects from the generalized linear mixed model (GLMM) for junior physicians’ assessment accuracy

Effect	F	df_1_	df_2_	*p-*value
Condition	9.802	4	5828	< 0.001
Period	0.157	4	5828	0.960
Task	43.089	3	5828	< 0.001

GLMM with binomial distribution and logit link function. df_1_ = numerator degrees of freedom; df_2_ = denominator degrees of freedom. Condition refers to the five assistance modes: (1) LLMs-free (independent decision-making), (2) DeepSeek-Chat, (3) DeepSeek-R1, (4) DeepSeek-Chat + KB, and (5) DeepSeek-R1 + KB. Period refers to the five assessment rounds in the Latin square crossover design (1–5), included to test for potential order or learning effects. Task refers to the four clinical assessment tasks: (1) ASA score, (2) RCRI score, (3) RCRI risk stratification, and (4) surgical tolerance judgment

**Table 5:** Pairwise comparisons of assistance conditions against the DeepSeek-R1 + KB reference group

Comparison	OR	95% CI	*p-*value
R1 + KB vs. LLMs-free	1.58	1.16–2.15	0.004
R1 + KB vs. Chat	2.51	1.86–3.39	< 0.001
R1 + KB vs. R1	1.82	1.34–2.47	< 0.001
R1 + KB vs. Chat + KB	1.40	1.02–1.90	0.035

OR = odds ratio; CI = confidence interval. P-values were derived from the GLMM fixed coefficients

## Discussion

The primary outcome of the study indicates that the DeepSeek-R1 model with chain-of-thought reasoning capability exhibits significantly superior comprehensive efficacy to the DeepSeek-Chat model in the perioperative assessment of total joint replacement. The study found that the R1 series demonstrates higher logical rigor and diagnostic accuracy in key clinical tasks such as RCRI scoring and risk stratification, surgical tolerance assessment, and perioperative core risk prediction. By integrating a professional knowledge base based on RAG technology, the DeepSeek-R1 + KB model further enhances its evidence-based decision support capability in complex clinical scenarios and can significantly improve the clinical decision-making accuracy of junior physicians. This result not only verifies the feasibility of applying LLMs in the orthopedic specialty but also reveals that the underlying structured logical reasoning module of the model is the core factor determining the efficiency of clinical knowledge translation and the value of auxiliary decision-making [27].

Importantly, the practical value of the DeepSeek-R1 + KB model in arthroplasty lies not only in its scoring accuracy but also in its ability to support arthroplasty-specific clinical decisions. For example, by accurately identifying patients with high RCRI scores or poor surgical tolerance, the model can alert junior physicians to initiate preoperative optimization (e.g., cardiac evaluation or glycemic control) before elective joint replacement. In terms of infection risk mitigation, although the Chat series also performed well in predicting infection, the R1 series provided more consistent reasoning regarding the need for preoperative decolonization based on patient risk factors. For VTE prophylaxis planning, the high prediction accuracy for VTE suggests that the model can reliably recommend mechanical or pharmacological prophylaxis according to current arthroplasty guidelines. Furthermore, the model’s judgment on surgical tolerance directly informs postponement decisions—a critical but difficult task for junior physicians. In our human–machine collaboration experiment, junior physicians assisted by DeepSeek-R1 + KB showed a significant improvement in surgical tolerance judgment (from 76.0% to 88.0%), indicating that the model can effectively support decisions on whether to proceed with or postpone elective arthroplasty. Therefore, while the core metrics (ASA, RCRI) are general, their application in arthroplasty is highly specific and actionable.

The significant discrepancy in performance between the DeepSeek-Chat and DeepSeek-R1 models may be primarily attributed to their architectural differences [28]. Compared with DeepSeek-Chat, the DeepSeek-R1 model features an upgraded structured logical reasoning module. For tasks in orthopedic preoperative assessment that rely on explicit scoring rules and classification criteria (e.g., ASA scoring and RCRI scoring), this module enables more accurate rule decomposition, element matching, and result deduction, thereby markedly reducing judgment deviations caused by logical discontinuities. In contrast, the reasoning mechanism of DeepSeek-Chat is more oriented toward general text comprehension, lacking sufficient capability to interpret the rules of standardized clinical scoring tools, which easily leads to issues such as missed element identification and weight mismatching. In terms of decision-making style, the relatively high FNR of the DeepSeek-Chat series reflects its distinct conservative tendency in assessment, as it tends to recommend against surgery to avoid potential risks. On the contrary, the high TPR and low FNR of the DeepSeek-R1 series confirm its higher sensitivity in identifying potential surgical opportunities. This decision-making approach of DeepSeek-R1, based on logical deduction and rigorous integration of evidence-based data, is more aligned with the thinking habits of clinical experts. However, in this retrospective study, the “gold standard” for surgical tolerance was defined as the actual surgical status documented in the medical records (whether the patient underwent surgery). We acknowledge that this is an imperfect reference standard, as surgery may have been postponed for non-medical reasons or patients may have undergone surgery despite high medical risk. However, based on the available retrospective data, we were unable to systematically capture these non-medical factors. Therefore, the reported accuracy of model-based surgical tolerance judgment should be interpreted with caution.

Challa et al. evaluated the correlation between ASA scores assigned by ChatGPT and those determined by anesthesiologists in pediatric surgical patients, reporting a correlation ranging from 66 to 67% [29]. The findings of this study are consistent with theirs: the accuracy of ASA scoring by all four model groups was below 60%, with no statistically significant differences observed among them. However, the low exact-match accuracy for ASA scoring (all models < 60%) should not be interpreted solely as poor model performance. Our interobserver analysis demonstrated only moderate agreement among anesthesiologists, indicating that the reference standard itself has substantial subjectivity. In terms of RCRI scoring and its corresponding risk stratification, the superior performance of the R1 series can be ascribed to its enhanced accuracy in parsing standardized scoring rules, whereas the low accuracy of the Chat series stems from its ambiguous understanding of such rules. The lack of performance improvement after knowledge base integration may be related to the limited demand for unstructured knowledge in RCRI assessment, or even interference caused by knowledge redundancy. Although DeepSeek-R1 + KB exhibited statistically significant advantages in clear decision-making objectives such as surgical tolerance, its maximum accuracy of 58.6% still indicates that LLMs can only serve as auxiliary tools for physicians at the current stage. When addressing complex clinical decisions, human intervention must be maintained to compensate for the performance bottlenecks of models, as they cannot fully replace physicians’ individualized decision-making.

This study revealed that the impact of knowledge base integration varies across different model versions: the overall performance of the DeepSeek-R1 model was slightly improved after integrating the knowledge base, which is consistent with the conclusions of Chen et al. [12]; in contrast, the overall performance of the DeepSeek-Chat model declined after integration. The potential reasons for this phenomenon are as follows. First, after integrating the knowledge base, although the DeepSeek-Chat model can retrieve more factual information through the knowledge base, it may merely “reproduce” guideline content when faced with complex medical record information, without achieving deep logical alignment between guidelines and medical records. In contrast, the DeepSeek-R1 model incorporates Chain-of-Thought (CoT) reasoning, which actively verifies the consistency between guideline provisions and patients’ actual clinical data; this proactive reasoning capability enables DeepSeek-R1 to utilize external knowledge more effectively. Second, when a large volume of guideline texts is input into the model via RAG, the DeepSeek-Chat model may struggle to accurately filter out interfering information from lengthy content. The additional guideline information becomes “noise” for the model, diverting its attention from core medical record data and thereby disrupting decision-making logic and reducing reasoning scores. This finding suggests that in the construction of clinical decision support systems, simply expanding the scale of the knowledge base does not necessarily translate into improved decision-making quality; instead, the reasoning capability of the model is the core variable determining the knowledge conversion rate.

In surgical risk prediction, the DeepSeek-R1 basic model and DeepSeek-R1 + KB model achieved 100% accuracy in predicting core perioperative risks of hip and knee arthroplasty, such as “Infection” and “VTE”. This high accuracy is closely related to the abundant guideline evidence available for these risks in joint replacement surgery. We also found that the prediction accuracy of all models for “other” risk categories was below 35%, which is consistent with the well-known limitation of LLMs in diagnosing rare diseases [30]. The core reasons may include the following: first, the “other” risk category encompasses a series of complications with low incidence rates and diverse clinical manifestations. Unlike “Infection” or “VTE”, which have clear keywords and strong semantic relevance, “other” risks often lack characteristic clinical labels, making it difficult for models to extract subtle features sufficient to trigger decision-making from complex medical record texts. Second, although this study incorporated 21 core perioperative guidelines, these guidelines primarily focus on mainstream complications. When confronted with rare risks not covered by guidelines or complex comorbidities involving multiple factors, even the DeepSeek-R1 model, which has strong reasoning capabilities, cannot achieve accurate prediction without external evidence-based support. This explains why model performance was significantly improved in routine risk items after knowledge base integration, but no breakthrough was made in the “other” risks. This result provides an important insight for “human–machine collaboration”: LLMs have the potential to replace or even surpass junior physicians in standardized and procedural risk screening, effectively reducing the rate of missed diagnoses. However, in the assessment of atypical, rare, and complex intertwined risks, the experiential intervention of human physicians remains an insurmountable safety barrier.

In terms of model performance evaluation, the DeepSeek-R1 + KB group achieved the highest accuracy in all three indicators, namely it exhibited the optimal performance in comprehension, retrieval, and reasoning. In addition, the reasoning capability of all models (with a maximum accuracy of 66.3%) was inferior to their retrieval capability (with a minimum accuracy of 68.2%), which is consistent with the finding of Kim et al. that current state-of-the-art LLMs often lack commonsense medical reasoning capability and are prone to generating hallucinations [31]. Preoperative assessment in orthopedics requires logical deduction based on multi-source data, and insufficient reasoning capability of models may lead to decision-making biases. In the evaluation of the models’ clinical reference value, senior physicians rated all models at a moderate level, but they showed a stronger preference for the DeepSeek-R1 + KB and DeepSeek-R1 models. A possible explanation is that senior physicians possess rich clinical experience, and their core requirements for models are “rigorous logical reasoning” and “supplementation of guideline-based evidence”. Therefore, models with high-level reasoning capability and integrated knowledge bases are more capable of meeting their needs for validating decision-making rationale.

This study verified the improvement effect of DeepSeek models on the preoperative assessment decisions of junior orthopedic physicians via a balanced experimental design and GLMM. The GLMM results further substantiate the auxiliary value of DeepSeek-R1 + KB. After adjusting for physician variability and patient case mix, the model remained significantly superior to LLMs-free (independent decision-making) (OR = 1.58, *p* = 0.004), as well as the DeepSeek-R1 and Chat series. This confirms that the benefit of the DeepSeek-R1 + KB combination is not merely a reflection of case difficulty or physician skill, but represents a genuine enhancement in decision support. Importantly, no significant period effect was detected (*p* = 0.960), indicating that junior physicians did not exhibit learning or fatigue effects across the five assessment rounds. This supports the validity of the Latin square crossover design and rules out carryover contamination between conditions. The significant task effect (*p* < 0.001), with ASA scoring being the most difficult, is consistent with the well-recognized subjectivity of ASA classification reported in previous studies, and further validates the model’s performance on objectively defined tasks such as RCRI scoring. The above results indicate that the DeepSeek-R1 + KB model can serve as a potential auxiliary tool for junior doctors to assess the feasibility of surgeries during their clinical practice.

The safety implications of these findings warrant explicit consideration. In this study, even the best-performing model (DeepSeek-R1 + KB) exhibited an FPR of 45.7% and a FNR of 40.9% for surgical tolerance judgment. In the context of elective arthroplasty, a false positive recommendation (“tolerable” when surgery is actually unsafe) could lead to proceeding with surgery in a patient with incomplete medical optimization—potentially resulting in catastrophic perioperative complications, including major adverse cardiac events, prosthetic joint infection, or even death. Conversely, a false negative recommendation (“postpone” when surgery is actually appropriate) could lead to unnecessary surgical delay, prolonging pain, functional decline, and deterioration in quality of life for patients with end-stage osteoarthritis [32]. Junior physicians are particularly vulnerable to automation bias—the tendency to over-rely on algorithmic outputs without sufficient independent verification—which may inadvertently diminish their clinical reasoning development over time. Therefore, we strongly emphasize that the DeepSeek-R1 + KB system should be deployed exclusively within a human-in-the-loop framework, wherein the model serves as a supplementary educational and checklist-style prompting tool rather than a standalone decision-maker. A senior physician must retain final authority over all surgical decisions, with the ability to override model recommendations. Prospective validation with real-time safety monitoring remains essential before any clinical translation can be responsibly considered.

This study has several limitations. This study was based on single-center retrospective medical record data of joint replacement surgery, which may be subject to biases related to clinical practice habits and disease spectrum; its generalizability to medical institutions of different levels and various disease types needs to be further verified through multicenter studies. Secondly, the use of actual surgery status as the reference standard for surgical tolerance is a major limitation. This may have led to error classification bias, as non-medical reasons for postponement could not be identified from the retrospective records. Future prospective studies should incorporate expert panel adjudication or postoperative complication data to validate true surgical tolerance. Additionally, the knowledge base only integrated guidelines related to perioperative management, without incorporating diverse academic resources such as orthopedic textbooks and expert consensus statements, which may restrict the model’s ability to address specialized orthopedic scenarios.

## Conclusion

The DeepSeek-R1 model has demonstrated favorable auxiliary value in the perioperative assessment of joint replacement. Its integration with the knowledge base scheme exhibits excellent performance in risk prediction, which can provide a reference for physicians’ decision-making. Nevertheless, although the model shows promising performance in identifying core complications, it still has limitations in handling subjective assessment indicators as well as atypical and rare risks. This suggests that LLMs should be positioned as clinical auxiliary tools rather than standalone decision-making systems at the current stage. As a cost-effective artificial intelligence solution, the DeepSeek-R1 series models provide a new technical pathway for individualized assessment under the framework of precision medicine. In the future, through the fusion of multimodal data and continuous optimization of specialized knowledge bases, it is expected to further enhance their application efficacy in complex clinical scenarios. Meanwhile, it is necessary to conduct prospective validation, external testing, and safety evaluation before clinical implementation.

## Supplementary Information

Supplementary Material 1.

Supplementary Material 2.

Supplementary Material 3.

Supplementary Material 4.

## Data Availability

The datasets used and/or analyzed during the current study are available from the corresponding author on reasonable request.

## Abbreviations

AI: Artificial Intelligence
ANOVA: Analysis of Variance
ASA: American Society of Anesthesiologists
BMI: Body Mass Index
CoT: Chain-of-Thought
DECIDE-AI: Developmental and Exploratory Clinical Investigations of Decision-support systems driven by Artificial Intelligence
FN: False Negative
FNR: False Negative Rate
FP: False Positive
FPR: False Positive Rate
ICIO: Instruction-Context-Input–Output
KB: Knowledge Base
LLM: Large Language Model
MI: Myocardial Infarction
RAG: Retrieval-Augmented Generation
RCRI: Revised Cardiac Risk Index
STROCSS: Strengthening the Reporting of Cohort Studies in Surgery
TN: True Negative
TNR: True Negative Rate
TP: True Positive
TPR: True Positive Rate
VTE: Venous Thromboembolism
