# Supplementary material for: Evaluation of DeepSeek with a RAG-based knowledge base in preoperative assessment for arthroplasty to improve the decision-making accuracy of junior physicians
Source: Arthroplasty. 2026 Oct 13;8:77. doi: 10.1186/s42836-026-00437-z (PMC13660083; doi:10.1186/s42836-026-00437-z)
Supplement: Supplementary file 1 [file 42836_2026_437_MOESM1_ESM.docx]

**Supplement 1. General knowledge dataset compiled from PubMed and UpToDate.**

**PubMed**

1. K. Uzel, İ. Azboy, J. Parvizi, Venous thromboembolism in orthopedic surgery: global guidelines, Acta orthopaedica et traumatologica turcica, 57 (2023) 192.

2. D. Grits, A. Kuo, A.J. Acuña, L.T. Samuel, A.F. Kamath, The association between perioperative blood transfusions and venous thromboembolism risk following surgical management of hip fractures, Journal of Orthopaedics, 34 (2022) 123-131.

3. R.K. Jain, M.D. Porat, G.G. Klingenstein, J.J. Reid, R.E. Post, S.D. Schoifet, The AAHKS clinical research award: liposomal bupivacaine and periarticular injection are not superior to single-shot intra-articular injection for pain control in total knee arthroplasty, The Journal of arthroplasty, 31 (2016) 22-25.

4. M.J. Kirwan, Z.R. Diltz, D.T. Dixon, C.A. Rivera-Peraza, C.J. Gammage, W.M. Mihalko, J.W. Harkess, J.L. Guyton, J.R. Crockarell, M.C. Ford, The AAHKS Clinical Research Award: Extended postoperative oral tranexamic acid in total knee arthroplasty: a randomized controlled pilot study, The Journal of Arthroplasty, 39 (2024) S13-S17.

5. J. Arraut, J. Thomas, C.T. Oakley, O.S. Barzideh, J.C. Rozell, R. Schwarzkopf, The AAHKS Best Podium Presentation Research Award: A second dose of dexamethasone reduces postoperative opioid consumption and pain in total joint arthroplasty, The Journal of Arthroplasty, 38 (2023) S21-S28.

6. A. Sigmund, L.A. Russell, Optimizing rheumatoid arthritis patients for surgery, Current rheumatology reports, 20 (2018) 48.

**UpToDate**

1. Complications of total hip arthroplasty

Link:http://60.190.199.67:8282/contents/complications-of-total-hip-arthroplasty?search=Complications%20of%20total%20hip%20arthroplasty&source=search_result&selectedTitle=1~148&display_rank=1

2. Complications of total knee arthroplasty

Link:http://60.190.199.67:8282/contents/zh-Hans/complications-of-total-knee-arthroplasty?search=Complications%20of%20total%20knee%20arthroplasty&source=search_result&selectedTitle=1~131&usage_type=default&display_rank=1

3. Evaluation of perioperative pulmonary risk

Link:http://60.190.199.67:8282/contents/zh-Hans/evaluation-of-perioperative-pulmonary-risk?search=Evaluation%20of%20perioperative%20pulmonary%20risk&source=search_result&selectedTitle=1~150&usage_type=default&display_rank=1

4. Overview of the principles of medical consultation and perioperative medicine

Link:http://60.190.199.67:8282/contents/overview-of-the-principles-of-medical-consultation-and-perioperative-medicine

5. Perioperative anaphylaxis: Recognition and emergency management

Link:http://60.190.199.67:8282/contents/perioperative-anaphylaxis-recognition-and-emergency-management?search=Perioperative%20anaphylaxis%3A%20Recognition%20and%20emergency%20management&source=search_result&selectedTitle=1~150&display_rank=1

6. Perioperative blood management: Strategies to minimize transfusions

Link:http://60.190.199.67:8282/contents/perioperative-blood-management-strategies-to-minimize-transfusions?search=Perioperative%20blood%20management%3A%20Strategies%20to%20minimize%20transfusions&source=search_result&selectedTitle=1~150&display_rank=1

7. Perioperative glycemic management in adults with diabetes mellitus

Link:http://60.190.199.67:8282/contents/perioperative-glycemic-management-in-adults-with-diabetes-mellitus?search=Perioperative%20gly%20cemic%20management%20in%20adults%20with%20diabetes%20mellitus&source=search_result&selectedTitle=1~150&display_rank=1

8. Perioperative management of heart failure in patients undergoing noncardiac surgery

Link:http://60.190.199.67:8282/contents/perioperative-management-of-heart-failure-in-patients-undergoing-noncardiac-surgery?search=Perioperative%20management%20of%20heart%20failure%20in%20patients%20undergoing%20noncardiac%20surgery&source=search_result&selectedTitle=1~150&display_rank=1

9. Perioperative management of hypertension

Link:http://60.190.199.67:8282/contents/perioperative-management-of-hypertension?search=Perioperative%20management%20of%20hypertension&source=search_result&selectedTitle=1~150&display_rank=1

10. Perioperative medication management

Link:http://60.190.199.67:8282/contents/perioperative-medication-management?search=Perioperative%20medication%20management&source=search_result&selectedTitle=1~93&display_rank=1

11. Perioperative neurocognitive disorders in adults: Risk factors and mitigation strategies

Link:http://60.190.199.67:8282/contents/perioperative-neurocognitive-disorders-in-adults-risk-factors-and-mitigation-strategies?search=Perioperative%20neurocognitive%20disorders%20in%20adults%3A%20Risk%20factors%20and%20mitigation%20strategies&source=search_result&selectedTitle=1~150&display_rank=1

12. Preoperative evaluation and perioperative management of patients with rheumatic diseases

Link:http://60.190.199.67:8282/contents/preoperative-evaluation-and-perioperative-management-of-patients-with-rheumatic-diseases?search=Preoperative%20evaluation%20and%20perioperative%20management%20of%20patients%20with%20rheumatic%20diseases&source=search_result&selectedTitle=1~150&display_rank=1

13. Preoperative evaluation for noncardiac surgery in adults

Link:http://60.190.199.67:8282/contents/preoperative-evaluation-for-noncardiac-surgery-in-adults?search=Preoperative%20evaluation%20for%20noncardiac%20surgery%20in%20adults&source=search_result&selectedTitle=1~150&display_rank=1

14. Smoking or vaping: Perioperative management

Link:http://60.190.199.67:8282/contents/smoking-or-vaping-perioperative-management?search=Smoking%20or%20vaping%3A%20Perioperative%20management&source=search_result&selectedTitle=1~150&display_rank=1

15. Surgical risk and the preoperative evaluation and management of adults with obstructive sleep apnea

Link:http://60.190.199.67:8282/contents/surgical-risk-and-the-preoperative-evaluation-and-management-of-adults-with-obstructive-sleep-apnea?search=Surgical%20risk%20and%20the%20preoperative%20evaluation%20and%20management%20of%20adults%20with%20obstructive%20sleep%20apnea&source=search_result&selectedTitle=1~150&display_rank=1
