# Supplementary material for: Evaluation of DeepSeek with a RAG-based knowledge base in preoperative assessment for arthroplasty to improve the decision-making accuracy of junior physicians
Source: Arthroplasty. 2026 Oct 13;8:77. doi: 10.1186/s42836-026-00437-z (PMC13660083; doi:10.1186/s42836-026-00437-z)
Supplement: Supplementary file 2 [file 42836_2026_437_MOESM2_ESM.docx]

**Supplement 2. Specific evaluation criteria and grading basis for each dimension**

**Evidence of Correct Comprehension:** Yes/No

**Evidence of Correct Retrieval:** Yes/No

**Evidence of Correct Reasoning:** Yes/No

**Correctness: Evaluate the accuracy and reliability of the answers in terms of facts, logic and reasoning.**

1. **Highly Incorrect:** The answer contains core factual errors, serious logical fallacies, or completely wrong conclusions. The information is misleading or even harmful.
2. **Somewhat Incorrect:** The main body of the answer is wrong, or although part of the information is correct, it is mixed with critical errors that render the overall conclusion untrustworthy.
3. **Moderately Correct:** The answer is basically on the right track, but has some imprecise factual details, inappropriate examples, or minor logical flaws that do not affect the core argument.
4. **Mostly Correct:** The answer is basically accurate and reliable in terms of facts and logic, with credible information sources. It only has very minor, trivial flaws.
5. **Completely Correct:** The answer is completely accurate in facts, data, and logical reasoning, with rigorous argumentation and reliable conclusions that stand up to scrutiny and verification.

**Helpful: The degree to which the response directly addresses the user's needs and provides effective help.**

1. **Completely Unhelpful:** The response is completely irrelevant, incorrect, or cannot be used to solve the problem at all.
2. **Not Very Helpful:** The response solves only a small part of the problem, and its practical value is very low.
3. **Somewhat Helpful:** The response covers the main points, but does not fully solve the problem.
4. **Helpful:** The response is clear and concise, and effectively solves the user's core problem.
5. **Extremely Helpful:** The response is timely; it uses additional resources (if applicable) to provide a thorough solution, significantly improving the efficiency of problem-solving.

**Completeness: The degree to which the response fully covers the question requirements and provides comprehensive information.**

1. **Incomplete:** The response is seriously missing key information, or only covers the most basic content.
2. **Not Very Complete:** The response covers most of the main points, but key details are missing.
3. **Somewhat Complete:** The response covers the main topic, but some parts are not detailed enough.
4. **Complete:** The response covers all key information, and the content is clear and thorough.
5. **Extremely Complete:** The response is comprehensive; it covers the "core + expansion" content and addresses all related issues.

**Adaptiveness: The degree to which the response adapts to user needs, scenarios, or usage habits (e.g., tone, complexity).**

1. **Not Adaptive:** The response is completely rigid and does not consider the user's scenario or usage habits.
2. **Not Very Adaptive:** The response considers part of the user's scenario, but there are obvious inconsistencies.
3. **Somewhat Adaptive:** The response roughly fits the user's needs, but lacks personalization or flexibility.
4. **Adaptive:** The response adjusts its content and form according to the scenario, and basically meets the user's preferences.
5. **Highly Adaptive:** The response is fully personalized; it dynamically adapts to the user's needs/scenario (e.g., supplements incomplete information in a timely manner).

**Case**

**Gender:** Female

**Age:** 74 years old

**Marital Status:** Married

**Occupation:** Retired

**History Informant:** Patient herself

**Reliability:** Reliable

**Chief Complaint:**

Bilateral knee joint pain for over 10 years, with the right side being more severe.

**History of Present Illness**:

The patient developed insidious bilateral knee pain more than 10 years ago, which was exacerbated by weight-bearing activities and completely relieved by rest. The pain was accompanied by joint stiffness, without joint clicking or rigidity. She visited a local hospital and was diagnosed with knee osteoarthritis, receiving conservative pharmacotherapy and intermittent intra-articular injections of the bilateral knees, with the last injection administered 7 years prior. She occasionally experienced pain in the bilateral interphalangeal joints and lumbodorsal discomfort, with no pain in the bilateral hip joints or radiating pain in the lower extremities. Currently, the patient presents with a limping gait, and knee pain has impaired her quality of daily life. She was admitted to our hospital for further management. Anteroposterior radiographs of the bilateral knees taken at an external hospital in May 2020 revealed joint space narrowing and osteophyte formation in both knees. A diagnosis of bilateral knee osteoarthritis was made, and the patient was admitted to our department.

Since the onset of symptoms, the patient has maintained a normal mental state and appetite, with no significant weight change and normal sleep quality. She denies a history of dental caries, recent oral surgical procedures, recent urinary frequency/dysuria, recent fever, recent cough with expectoration, and recent abdominal pain with diarrhea.

**Past Medical History:**

The patient’s general health condition is fair. She has a 30-year history of hypertension, with self-reported well-controlled blood pressure. Renal impairment and hyperuricemia were detected during a physical examination at our hospital in 2019, for which she received conservative pharmacotherapy. Thirteen years ago, she presented with dizziness and bilateral lower limb weakness at an external hospital, where she was diagnosed with cerebral infarction and achieved symptom improvement after pharmacotherapy. Six years ago, she experienced a recurrence of dizziness and bilateral lower limb weakness triggered by elevated blood pressure, was re-diagnosed with cerebral infarction at an external hospital, and responded well to subsequent pharmacotherapy. She was evaluated at Beijing Friendship Hospital for exertional dyspnea, and a stress-rest myocardial perfusion SPECT scan suggested possible mild myocardial ischemia in the anterior wall and apical segment of the left ventricle. She denies a history of chronic diseases including coronary heart disease and diabetes mellitus, as well as infectious diseases such as viral hepatitis, tuberculosis, typhoid fever, and malaria. She underwent surgical resection for right breast cancer 3 years ago, followed by regular postoperative chemotherapy; a follow-up examination at our hospital in December 2020 showed no obvious abnormalities. She denies a history of major trauma or blood transfusion, and has no known drug or food allergies. Her vaccination history is unclear.

**Current Medications:**

Losartan Potassium and Hydrochlorothiazide Tablets: 1 tablet, once daily (qd)

Lercanidipine Hydrochloride Tablets: 1 tablet, twice daily (bid)

Arotinolol Hydrochloride Tablets: 1 tablet, once daily (qd)

Atorvastatin Calcium Tablets: 1 tablet, once daily (qd)

Aspirin Enteric-coated Tablets: 1 tablet, once daily (qd)

Isosorbide Mononitrate Tablets: 1 tablet, twice daily (bid)

Coated Aldehyde Oxystarch Capsules: 8 capsules, three times daily (tid)

Piperaquine Phosphate and Artesunate Tablets: 4 tablets, three times daily (tid)

Febuxostat Tablets: 1 tablet, once daily (qd)

Peiyuan Tongnao Capsules: 3 capsules, three times daily (tid)

Anastrozole Tablets: 1 tablet, once daily (qd)

Calcium Carbonate and Vitamin D3 Tablets: 1 tablet, once daily (qd)

Celecoxib Capsules: 2 capsules, twice daily (bid)

**Personal History:**

The patient was born and raised in her native place, with no history of long-term residence elsewhere. She denies exposure to epidemic areas or contaminated water sources, as well as contact with hazardous chemicals or radioactive substances. She has no habits such as smoking or drinking.

**Obstetric and Gynecologic History:**

Widowed; she has two daughters, both in good health. Her last menstrual period occurred at the age of 50, with no abnormal vaginal bleeding or discharge after menopause.

**Family History:**

She denies a family history of similar diseases, as well as familial psychiatric disorders, tumors, or hereditary diseases.

**Physical Examination:**

**Vital Signs:** Temperature (T): 36℃; Pulse (P): 70 beats/min; Respiratory Rate (R): 20 breaths/min; Blood Pressure (BP): 148/79 mmHg

**General Appearance:** Well-developed and well-nourished; conscious and alert; in a normal spontaneous position; with a calm facial expression; cooperative with physical examination

**Skin and Mucous Membranes:** No jaundice, petechiae, or ulcerations observed; no palpable enlarged superficial lymph nodes

**Head and Neck:** Normocephalic, no deformities, tenderness, masses, or nodules; no eyelid edema or ptosis; palpebral conjunctiva clear, no congestion, hemorrhage, pallor, or edema; sclera non-icteric; bilateral pupils equal in size and round, with brisk light reflexes; no abnormal discharge from the ears or nose; no tenderness over the mastoid processes or paranasal sinuses; bilateral hearing intact; lips pink and moist; oral mucosa free of ulcers or leukoplakia; pharynx non-congested; bilateral tonsils not enlarged; tongue of normal size, protrudes midline without tremor; neck soft and supple, no resistance; jugular veins non-distended; trachea midline; bilateral thyroid glands not enlarged; no vascular bruits auscultated over the neck

**Chest:** A healed surgical scar is visible on the chest wall; thoracic cage symmetric; bilateral chest expansion symmetric; tactile fremitus symmetric bilaterally; no pleural friction rub; breath sounds clear bilaterally, with no rhonchi or crackles; precordium non-prominent and non-depressed; cardiac silhouette normal in size; rhythm regular; no pathological murmurs auscultated over the cardiac valve areas; peripheral vascular signs negative

**Abdomen:** Soft, non-tender, with no rebound tenderness; bowel sounds present at a rate of 3 times per minute; liver and spleen not palpable below the costal margin or xiphoid process; no tenderness at McBurney’s point or bilateral ureteral points; Murphy’s sign negative; shifting dullness negative

**Spine and Extremities:** Spine straight, no deformities or tenderness; joint movements of the extremities unrestricted; no edema of the lower extremities; dorsalis pedis artery pulses palpable and normal bilaterally

**Genitourinary and Rectal Examination:** Not performed

**Neurological Examination:** Physiological reflexes present; pathological reflexes not elicited

**Orthopedic Special Examination:**

**Gait:** Mild limping gait; bilateral genu varum deformity

**Hands:** No obvious redness or swelling of the bilateral interphalangeal and metacarpophalangeal joints; flexion deformity of the proximal interphalangeal joint of the left third finger

**Spine:** Normal physiological curvature; no tenderness over the spinous processes; no obvious scoliosis

**Pelvis:** No obvious tilting

**Muscles:** No significant atrophy of the bilateral quadriceps femoris muscles; no obvious varicose veins of the lower extremities

**Knee Joints:** No obvious swelling of the bilateral knees

Right Knee: Tenderness (+) over the medial joint space; hyperextension test (+); hyperflexion test (+); McMurray test (+); grind test (+); valgus/varus stress tests (-); anterior/posterior drawer tests (-); patellar tap test (-); Range of Motion (ROM): 0° (extension) - 120° (flexion)

Left Knee: Tenderness (+) over the medial joint space; tenderness (-) over the lateral joint space; hyperextension test (+); hyperflexion test (+); McMurray test (+); patellar grind test (+); anterior/posterior drawer tests (-); valgus/varus stress tests (-); patellar tap test (-); Range of Motion (ROM): 0° (extension) - 120° (flexion)

**Hip Joints:** Bilateral hip joints with full range of motion, no obvious deformities

**Muscle Strength and Tone:** Muscle strength of the bilateral iliopsoas and quadriceps femoris muscles graded as 5/5; normal muscle tone of the lower extremities

**Sensory Function:** Symmetric and normal superficial and deep sensation in the bilateral extremities

**Neurological Reflexes:** Normal bilateral knee reflexes; Babinski sign (-) bilaterally; straight leg raising test (-) bilaterally

**Vascular Examination:** Palpable and normal dorsalis pedis artery pulses bilaterally

Limb Length Measurement:

**Relative Length of Lower Extremities:** Left 75 cm, Right 75 cm

**Absolute Length of Lower Extremities:** Left 69 cm, Right 69 cm

**Limb Circumference Measurement:**

10 cm above the superior pole of the patella: Left 39 cm, Right 39 cm

10 cm below the tibial tuberosity: Left 32 cm, Right 32 cm

**Auxiliary Examinations:**

Anteroposterior radiographs of bilateral knees (external hospital, May 2020): Joint space narrowing and osteophyte formation in both knees.

Coronary CT Angiography (21 February 2021, result pending): Right coronary artery dominance type. Findings include: (1) Moderate coronary artery calcification; (2) No definite stenosis of the left main coronary artery; mixed plaque in the proximal segment of the left anterior descending artery with moderate luminal stenosis; no definite stenosis of the left circumflex artery; multiple mixed plaques in the right coronary artery with moderate luminal stenosis in the proximal segment and focal mild stenosis in the middle and distal segments; (3) No significant abnormalities in the size of the bilateral ventricles; ventricular wall thickness approximately normal; no obvious vegetations on the mitral, tricuspid, or aortic valves.

Color Doppler Ultrasound of Carotid and Vertebral Arteries (19 February 2021): Bilateral carotid atherosclerosis with left carotid plaque formation; increased resistance in the right vertebral artery.

Transthoracic Echocardiogram (Department of Cardiology, 10 February 2021): Ascending aorta dilation; degenerative aortic valve disease; mild aortic regurgitation.

Routine Brain MRI (20 February 2021): Non-specific white matter changes in the bilateral frontal lobes and left parietal lobe subcortex; age-related cerebral changes; possible multiple perivascular spaces in the bilateral centrum semiovale, basal ganglia, and thalamus; nasal septum deviation. Clinical correlation is recommended; contrast-enhanced MRI is advisable if necessary.

Abdominal Ultrasound (Liver, Gallbladder, Pancreas, Spleen, Kidneys, 18 February 2021): Fatty liver; cholesterol crystals in the gallbladder wall; hyperechoic foci in the gallbladder, not excluding sediment-like gallstones; partial left renal pelvic dilatation.

**Admission Diagnoses:**

Severe bilateral knee osteoarthritis (right side more severe)

Essential hypertension

Coronary heart disease

Old cerebral infarction

Gallbladder stones

Chronic renal insufficiency

Hyperuricemia

Status post right breast cancer resection

**Question**

You are an attending orthopedic physician and are now required to conduct a preoperative assessment for a patient scheduled to undergo joint replacement surgery. Based on the uploaded patient medical record, you need to complete and output the following tasks:

1. ASA (American Society of Anesthesiologists) Score: Assign an ASA physical status classification and explain the rationale based on the patient’s overall health condition.
2. RCRI (Revised Cardiac Risk Index) Score and Risk Stratification: Calculate the RCRI score according to the medical record details and determine the corresponding cardiac risk grade.
3. Surgical Tolerance Assessment: Clearly state whether the patient can tolerate the surgery at present, or whether the surgery needs to be postponed, and list the adjustment targets (e.g., infection control, blood glucose/blood pressure management).

**Answer**

Based on the medical records provided, I will conduct a comprehensive preoperative assessment of this 74-year-old female patient from the perspective of an orthopedic physician:

**I.Surgical Risk Assessment and Perioperative Recommendations**

**Primary Surgical Risks**

High Cardiovascular Risks

Coronary heart disease (moderate coronary artery stenosis)

30-year history of hypertension

History of two episodes of old cerebral infarction

Possible mild myocardial ischemia

**Other Systemic Risks**

Renal insufficiency

Hyperuricemia

Gallbladder stones

Postoperative status of right breast cancer

Reduced physiological reserve due to advanced age

**Detailed Perioperative Recommendations**

**1. Medication Management Recommendations**

Aspirin: Discontinue 7 days preoperatively and switch to low-molecular-weight heparin for bridging therapy.

Antihypertensive drugs: Continue losartan potassium and hydrochlorothiazide tablets, lercanidipine hydrochloride tablets, and arotinolol hydrochloride tablets until the morning of surgery.

Statin: Continue atorvastatin administration.

Isosorbide mononitrate: Maintain continuous use.

Febuxostat: Continue to control serum uric acid levels.

Celecoxib: Discontinue 3–5 days preoperatively (nonsteroidal anti-inflammatory drugs impair platelet function).

Other medications: Adjust according to consultation opinions from relevant specialist departments.

**2. Required Further Auxiliary Examinations**

Cardiac function assessment:

Recent electrocardiogram (ECG)

Repeat echocardiography (to evaluate left ventricular ejection fraction [LVEF] and overall cardiac function)

Holter monitoring if necessary

Renal function assessment:

Serum creatinine, blood urea nitrogen (BUN), and estimated glomerular filtration rate (eGFR)

Urinalysis plus microalbumin measurement

Coagulation function: Coagulation panel (prothrombin time, activated partial thromboplastin time, fibrinogen, international normalized ratio)

Complete blood count (CBC), liver function tests, and electrolyte panel

Lower extremity vascular ultrasonography (to assess vascular patency and morphology)

**3. Mandatory Specialist Consultations**

Cardiology consultation: Evaluate cardiac risk and formulate perioperative management strategies.

Nephrology consultation: Assess renal function status and guide medication adjustment.

Anesthesiology consultation: Design an individualized anesthesia plan.

**II. ASA Physical Status Classification**

ASA Class III – A patient with severe systemic disease that limits activity but does not preclude work capability.

Rationale: The patient suffers from multiple severe systemic conditions, including hypertension, coronary heart disease, old cerebral infarction, and renal insufficiency. However, she is still able to maintain basic daily functional activities.

**III. Surgical Tolerance Evaluation**

**Surgery should be postponed for adequate preoperative optimization under the current conditions.**

**Rationale for Postponement**

1. **Suboptimal cardiovascular status**

Further evaluation and optimized treatment regimen are required by the cardiology team.

Blood pressure control needs further adjustment (admission blood pressure: 148/79 mmHg).

2. **Necessity of polypharmacy adjustment**

Bridging therapy is required for antiplatelet medications.

A sufficient washout period is needed for NSAID discontinuation.

3. **Need for definitive renal function assessment**

The current degree of renal impairment must be clearly defined.

4. **Comprehensive risk assessment**

As an elderly patient with multiple comorbidities, she requires multidisciplinary collaboration for preoperative optimization.

**Recommendations**

1. Complete all the required auxiliary examinations first.
2. Conduct consultations with relevant specialist departments and optimize the function of all affected systems.
3. Adjust the medication regimen, particularly the antiplatelet therapy strategy.
4. Reassess the optimal surgical timing after the function of all systems stabilizes.
5. Consider staged surgery, with priority given to the symptomatic right knee joint.

**Estimated optimization period: 1–2 weeks**

Only after thorough preoperative preparation and achievement of relatively stable function across all systems can the patient safely undergo joint replacement surgery.

**Subjective scoring**

**Evidence of Correct Comprehension:** Yes

**Evidence of Correct Retrieval:** Yes

**Evidence of Correct Reasoning:** No

**Correctness: 3 (Moderately Correct)**

**Helpful: 2 (Not Very Helpful)**

**Completeness: 2 (Not Very Complete)**

**Adaptiveness: 3 (Somewhat Adaptive)**
